# Supplementary material for: Metagenomic Sequencing to Analyze Composition and Function of Top-Gray Chalky Grain Microorganisms from Hybrid Rice Seeds
Source: Plants (Basel). 2023 Jun 18;12(12):2358. doi: 10.3390/plants12122358 (PMC10305155; doi:10.3390/plants12122358)
Supplement: Supplementary file 1 [file plants-12-02358-s001.zip › Table S1.pdf]

Table S1. Pre-processing results of sequencing data

Raw data (Mbp)	Clean data (Mbp)	Non-host data (Mbp)	Effective rate (%)
55,870.57	55,824.05	55,725.44	99.82
