# Supplementary material for: Metagenomic Sequencing to Analyze Composition and Function of Top-Gray Chalky Grain Microorganisms from Hybrid Rice Seeds
Source: Plants (Basel). 2023 Jun 18;12(12):2358. doi: 10.3390/plants12122358 (PMC10305155; doi:10.3390/plants12122358)
Supplement: Supplementary file 1 [file plants-12-02358-s001.zip › Table S2.pdf]

Table S2. Metagenome assembly

Scaffolds (bp)		Scaftigs (bp)		Open reading frames (ORFs)	
Total	468,836,453	Total	468,836,453	Total	570,029
Average	1,485.82	Average	1,486	Gene catalogue	250,918
N50	2,069.11	N50	2,069	Integrated gene catalog (IGC)	149,383
N90	634.33	N90	634	Integrated gene percent	59.53%
				GC percent	46.97%
